# Electricity in Neuralgic Diseases

**Published:** 1841-11

**Authors:** 


ELECTRICITY IN NEURALGIC DISEASES.

This subject is attracting much attention, and the medical journals of Europe abound in cases of neuralgia successfully treated by electricity. The forms of its application are various—as galvanism, the electric spark, shock, and aura, magnetism, electro-magnetism, and acupuncture with combined galvanism. Of all these methods, that of the electric-magnetic apparatus, with sponge directors, seems to us the most promising, whilst, at the same time, it is mild, steady, and entirely under the control of the operator as to intensity and duration. In some cases of neuralgia, where the ordinary plans of cure have proved unavailing, this holds out a dernier hope to patients which is worthy of resort. The disease appears to be more common in this country than in earlier times, and especially afflicts the citizens of the larger towns. Some such patients we have now under treatment, by the electric-magnetic machine, and the result of the trials we will lay before our readers in due season. Y.

